# Gastroesophageal Reflux Disease and Asthma: A Narrative Review

**DOI:** 10.7759/cureus.24917

**Published:** 2022-05-11

**Authors:** Xavier A Grandes, Ramya Talanki Manjunatha, Salma Habib, Sai Lahari Sangaraju, Daniela Yepez

**Affiliations:** 1 Research, Universidad Catolica Santiago de Guayaquil, Guayaquil, ECU; 2 Internal Medicine, Kempegowda Institute of Medical Sciences, Bangalore, IND; 3 Medicine and Surgery, Institute of Applied Health Sciences, Chittagong, BGD; 4 Research, People's Education Society (PES) Institute of Medical Sciences and Research, Kuppam, IND

**Keywords:** pharmacologic management, clinical correlation, pathophysiology, asthma, gastroesophageal reflux disease (gerd)

## Abstract

Gastroesophageal reflux disease (GERD) is defined by retrograde reflux of gastric contents to the esophagus leading to various signs and symptoms that range from heartburn/regurgitation to the development of extraesophageal respiratory syndromes like asthma. Although a cause-effect relationship has been proposed, evidence suggests that these two entities share a complex mechanism that may be reciprocal to each other. The understanding of the underlying mechanisms is imperial due to the fact that asthmatic patients may benefit from reflux therapy with subsequent improvement in pulmonary function testing and quality of life. This study has revised available literature in order to provide evidence for a nexus between GERD and asthma based on clinical correlation, pathophysiology, and pharmacologic management.

## Introduction and background

Gastroesophageal reflux disease (GERD) is a prevalent condition defined by a chronic retrograde movement of gastric content to the esophagus leading to a wide range of symptoms and complications in patients [1]. The prevalence of GERD in locations such as South America, Western Europe, and North America have the highest prevalence rates that range from 20% to 40% [2]. Risk factors such as female gender, obesity (body mass index >30), and advancing age have all been associated with an increase in GERD-related symptoms [3]. Both Caucasians and African American patients have a high incidence of GERD. Nevertheless, African Americans have a lower prevalence and a lower probability of the development of esophagitis [4]. The pathophysiology of GERD is a complex topic that involves several mechanisms that impair the protective capacity of the esophagus to overcome the reflux of acid, pepsin, duodenal bolus, and pancreatic enzymes [1]. The mechanisms implicated include motor anomalies, anatomical anomalies, and impaired resistance of the mucosa [1]. GERD-related symptoms arise due to lesions of the mucosa lining the esophagus and may manifest as esophageal (regurgitation and heartburn) or extraesophageal (chronic cough, asthma, dental erosions, laryngitis, and non-cardiac chest pain) findings [5-8]. These atypical manifestations of GERD, known as extraesophageal syndromes (EOS), have gained a lot of attention in the last few decades, both in the clinical setting and in medical literature and the role of GERD in the pathophysiology of numerous respiratory illnesses has become a topic of debate [3].

In 1966, a link was discovered between GERD and respiratory diseases, implicating GERD in the pathogenesis of asthma [9-11]. According to the findings of the extensive European ProGERD study, 4.8% of GERD patients have asthma and is considered the third most frequent EOS in North America, according to a comparable survey [10]. GERD is frequently cited in asthmatic patients. A systematic review of 28 studies determined the prevalence of gastroesophageal reflux symptoms in asthmatic patients to be 59.2% compared to 38.1% in controls [12]. The explanation behind bronchoconstriction due to acid reflux can be explained by three postulated mechanisms: increased bronchial reactivity, micro-aspiration of acid and other gastric contents into the upper airways, and increased vagal tone [12]. Other authors have suggested that the association between GERD and asthma can be explained by the "reflux theory," otherwise known as a direct mechanism and the "reflex theory" as an indirect mechanism [13,14]. However, an unresolved question remains, does asthma worsen GERD? or does GERD exacerbate asthma? Many factors have been identified that can contribute to GERD worsening in asthmatic patients; these include coughing and increased effort in respiration, hyperinflation of the lungs, contractions of the diaphragm, and increased pressure gradient across the lower esophageal sphincter [15,16]. Different types of medication used in asthma may promote reflux; these include theophylline, β-agonists, and corticosteroids [17]. Reciprocally, GERD should be suspected as the underlying cause of asthma in adult patients with an acute onset of asthma, negative family history, negative allergic component, unfavorable response to asthma medications, or postprandial worsening of symptoms [13]. The aim and objectives of this review were to provide an insight into the clinical correlation, pathophysiology, and pharmacologic management between GERD and asthma.

## Review

Mechanism of GERD and asthma: is there a link?

The cause-effect relationship between GERD and asthma is difficult to establish since it has been proven that either disease process can trigger the development of each other [18]. GERD-Induced asthma should be suspected in all patients who present at an adult age with new-onset asthma symptoms, poor asthma control even with proper medication, and heartburn or regurgitation preceding asthmatic event [18]. The symptoms of gastroesophageal reflux (GER), such as heartburn and regurgitation, affect a large percentage of asthma patients, and the absence of traditional symptoms does not rule out physiologic acid reflux in those who have a chronic cough [18]. A systematic review conducted in 2007 by Havemann et al. discovered that the average prevalence of GERD symptoms in asthma patients was 59.2%. However, the prevalence of asthma in patients with GERD was estimated to be 4.6% [12]. This could be interpreted as an underestimation due to the fact that a vast majority of patients may not present with the classic symptoms of reflux [12,18]. Additionally, a double-blind, placebo-controlled crossover study conducted in 1999 by Kiljander et al. found that pathologic GERD is present in 53% of asthmatic patients, and approximately one-third of these patients did not exhibit typical reflux symptoms [19]. According to these studies, there is a high prevalence of GERD in the asthmatic population which most often presents as clinically silent, and therefore reflux should be suspected even if patients do not complain of classic symptoms like heartburn and regurgitation [12,19]. The complex pathophysiology explaining the correlation between asthma and GERD is still a heavily debated topic [3]. However, two proposed mechanisms have been studied and are widely accepted; a “reflux theory” and a “reflex theory” [18].

The Reflux Theory

This theory refers to a direct mechanism by which gastroduodenal reflux (acid, pepsin, bile acids, and pancreatic enzymes) enter the esophagus and subsequently leads to microaspiration into the lungs and other parts of the pulmonary tree [20]. The aspiration of these contents can result in stimulation of the pharynx or larynx, consequently resulting in extraesophageal symptoms such as tracheal or bronchial cough reflex [3]. The chronic inflammation of lung tissue may lead to airway obstruction, poor gas exchange, acute lung injury, and severe acute respiratory distress syndrome [21]. These mechanisms consequently induce the liberation of proinflammatory cytokines from T-helper type 2 cells, leading to an increase in airflow resistance and inflammation [18]. Bronchoconstriction is induced by direct HCl intra-esophageal instillation via muscarinic (M3) receptors, which release acetylcholine [22]. This contributes to airway inflammation and the stimulation of smooth muscle contractions in the airways [22]. Infiltrates of macrophages, neutrophils, eosinophils, and lymphocytes are detected histologically in GERD-induced airway inflammation [23]. Several animal investigations have shown that the release of various interleukins increases tumor necrosis factor (TNF)-alpha production [23]. These findings correlate with a study conducted by Tuchman et al. in 1984, wherein 13 sedated adult cats, the brief latent airway response after acid infusion into the trachea or esophagus was compared quantitatively [24]. In all animals examined, a 0.05 ml infusion of 0.2 N HCl induced a 4.65-fold increase in total lung resistance compared to baseline (p < 0.005) and found that intratracheal acid infusion elicited a fast-adapting, pH-dependent, and vagally mediated response [24]. Human studies have also been performed and indicate the importance of the reflux theory. Jack et al. conducted a study in 1995 where tracheal and esophageal pH measurements over a 24-hour time frame were taken [25]. The results of this study found that in four patients with asthma and symptomatic GERD, 37 episodes of reflux that lasted more than five minutes were registered, and five of these were followed by a drop in tracheal pH from 7.1 (0.2) to 4.1 (0.4) and a drop in peak expiratory flow rate (PEFR) from 84 (16) l/min [25]. In addition, Pauwels et al. conducted a study in 2012 on 41 patients with cystic fibrosis, 15 controls, 29 asthmatic patients, and 28 patients with chronic cough [26]. The findings of this study aimed to assess gastroduodenal components in induced sputum. Results found that in eight of the 29 patients (28%) with asthma, there was a presence of bile acids in sputum [26]. Furthermore, the levels of bile acids were clearly related to the degree of lung function impairment in individuals with bile acid aspiration, implying that micro-aspiration had a negative effect [3]. In contrast, Mise et al. conducted a case-control study in the year 2010 to determine a direct measurement of lung pH in patients with GERD vs. a control group [27]. The findings of his study concluded that patients with GERD have an average pH in the lung of 5.13+/- 0.43 vs. 6.08 +/-0.39 (p=0.001) in controls and increased levels of LDH in bronchoalveolar aspirate [27]. When pH is 6 in vivo, an increase in lactate dehydrogenase (LDH) is a marker of greater cell and tissue damage, which reflects the direct damage of acid reflux in GERD [18]. 

The Reflex Theory

The reflex theory refers to a method otherwise known as an indirect process by which distal esophageal reflux induces stimulation of the vagus nerve leading to bronchoconstriction [3]. This method operates on the basis that the esophagus and tracheobronchial tree share an embryological origin and would therefore share similar innervation [18]. Consequently, this shared innervation could explain why stimuli in the distal esophagus lead to extraesophageal symptoms via vagus nerve reflexes [18]. A study conducted by Harding et al. in 1999 sought to examine the prevalence and severity of GERD in asthmatics with and without reflux symptoms as well as the correlation of respiratory symptoms with intra-esophageal acid [28]. A total of 199 asthmatics were analyzed; 164 (82%) had underlying reflux symptoms, 118/164 (72%) asthmatics with reflux symptoms had abnormal 24-h esophageal pH tests vs. 10/35 asthmatics without reflux symptoms. Among asthmatics with GER, esophageal acid was linked to 119 of 151 respiratory symptoms (78.8%) [28]. The findings by Harding et al. correlate with a study performed by Avidan et al., which aimed to evaluate asthmatics with gastroesophageal reflux, utilizing 24-H esophageal tracings to see if there was a temporal link between acid reflux and coughing or wheezing [29]. During the 24-h recording period, 53 asthmatics had five or more coughs, and 19 had three or more wheezes. In asthmatics with and without pulmonary symptoms, mean acid contact time was similar (12.2 {1.2}% vs. 10.4 {0.6}%). Acid reflux was linked to 46% and 48% of all coughs and wheezes, respectively [29]. One recent animal study conducted by Cheng et al. in 2014 aimed to clarify functional changes of bronchial smooth muscles isolated from guinea pigs in an animal model of GERD [30]. Results indicate that acid infusion into the lower esophagus, followed by microaspiration into the respiratory tract, causes airway hyperresponsiveness and overactive bronchial smooth muscle in guinea pigs [30]. Nonetheless, not all available literature data is consistent with this theory. A systematic review conducted by Field in 1999 aimed to analyze in asthmatic individuals the impact of both spontaneous and artificial gastroesophageal reflux (GER) on pulmonary function [31]. A total of 254 citations were identified, and 18 studies of GERD and acid perfusion in asthmatic individuals were included in the list. The forced expiratory volume in the first second (FEV1) and the mid expiratory rate did not change during acid perfusion or gastroesophageal reflux in trials with 97% and 94% of asthmatics, respectively, according to these findings, which contain data on 312 asthmatics [31]. These findings are consistent with a study conducted by Ekström and Tibbling, whose main goal was to elucidate if esophageal acid stimulation can cause clinically detectable bronchospasm in asthmatic patients and if such a reaction is connected to bronchial reactivity [32]. Results obtained demonstrated that in patients with moderate to severe asthma with GERD (n=8), daytime instillation of esophageal acid into the esophagus did not cause clinically significant bronchoconstriction, respiratory symptoms, or a rise in airway responsiveness. Furthermore, changes in FEV1 in response to esophageal acid stimulation were minor and inconsistent [32].

Other Proposed Mechanisms

**Figure 1:**
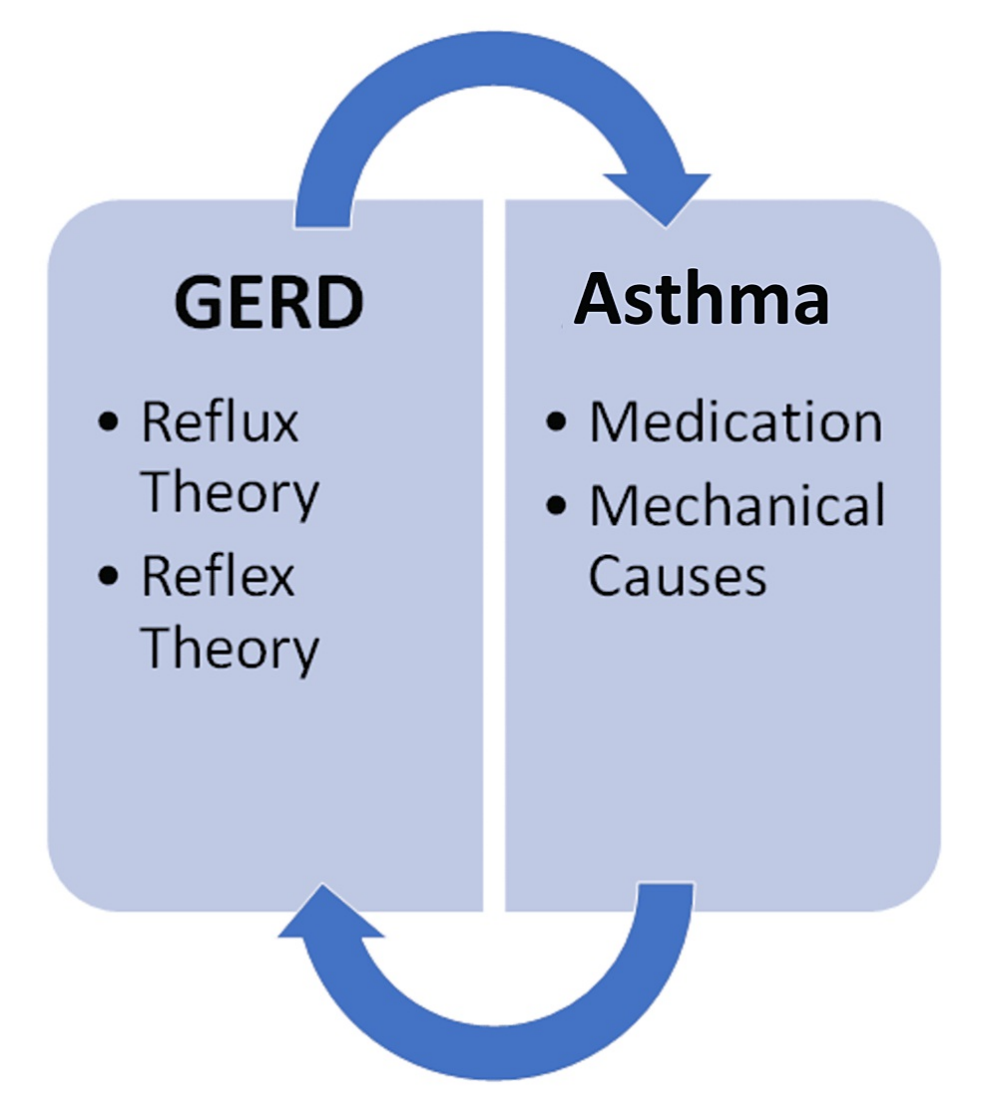
Proposed mechanisms between GERD and asthma. GERD: gastroesophageal reflux disease The image is created by the author (Xavier Grandes) of this study.

Although there is strong evidence that GERD can lead to the development of asthma, many authors have also theorized that asthma can reciprocally lead to worsening of GERD by two mechanisms: asthma medication and mechanical causes [18]. Anti-asthmatic drugs, such as beta 2 adrenergic receptor agonists or theophylline, are believed to cause the relaxation of smooth muscles, including the lower esophageal sphincter (LES) [18]. This was demonstrated by Ekstrom and Tibbling, who studied theophylline use and discovered a 24% increase in daytime reflux, as well as a 170% increase in heartburn and regurgitation sensations; interestingly, no LES measurements were taken in this investigation [33]. In the year 1991, Michoud et al. conducted a study to see if beta-adrenergic agonists cause or worsen gastroesophageal reflux in asthma patients [34]. The findings show that salbutamol did not affect the lower esophageal sphincter gradient pressure, peak esophageal contraction pressure, or even the number and duration of reflux episodes in asthma patients [34]. Mechanical changes are due to the underlying obstructive pattern of asthma, which causes an increase in negative intrathoracic pleural pressure [35]. This negative pressure induces an increase in diaphragmatic pressure generating a pressure gradient that facilitates the reflux of gastroduodenal content [35]. These findings were also consistent with Michoud et al., who found that patients with asthma have a higher lower esophageal sphincter pressure at rest, in contrast with healthy subjects [34]. A diagram depicting the interplay between these mechanisms can be seen below (Figure 1).

Prevalence of GERD symptoms in asthma

GERD clinical prevalence in asthmatic patients is widely variable, and it is estimated to be around 30-50% [3]. Havemann et al. conducted a systematic review in the year 2007 that analyzed 28 studies which determined that in asthma patients, the sample size weighted average prevalence of GERD symptoms was 59.2%, compared to 38.1% in controls [12]. However, individuals with GERD had a 4.6% asthma prevalence, whereas controls had a 3.9% prevalence. Interestingly, when the odds ratios were combined, the overall ratio for studies reporting the prevalence of GERD symptoms in subjects with asthma was 5.5 (95% CI, 1.9-15.8), and 2.3 (95% CI, 1.8-2.8) for studies evaluating the prevalence of asthma in GERD patients [12]. These findings are consistent with a cross-sectional study conducted by Field et al. in 1996 that sought to estimate the prevalence of symptomatic GERD in Asthmatics [36]. Among the asthmatics (n=109) vs. control groups (n=68, n=67), 77% had symptoms compatible with heartburn, 55% experienced regurgitations, and 24% presented difficulty swallowing [36]. Additionally, another cross-sectional study that correlates with these findings was performed by Gislason et al. in 2002, whose main objective was to estimate the association between nocturnal GERD symptoms, respiratory symptoms, sleep-disordered breathing, and asthma [37]. Of the 2661 subjects, the asthma population (n=459) presented with nocturnal GERD and esophagitis in approximately 40% of cases, in contrast to the random population sample (n=101), which was present in only 4.6% of cases [37]. Nevertheless, not all published studies have presented findings consistent with the prevalence of GERD symptoms in asthma. Bor et al. carried out a study in 2010 to determine the prevalence of GERD among asthmatics and patients with chronic obstructive pulmonary disease (COPD) [35]. Having employed a validated face-to-face questionnaire, the prevalence of GERD (defined as heartburn/regurgitation that presents once or more times per week) was present in 25.4% of asthmatics (n=308) vs. 19.4% in controls (n=694), respectively. Some patients presented with occasional symptoms of GERD (less than one week) in 21.2% of asthmatics vs. 27.0% of controls [35]. A related study by Shirai et al. in 2015 displayed similar findings. Among the 132 subjects with controlled asthma, it was determined that 29 patients (22.0%) had GERD [38]. These results seem to suggest that the prevalence of GERD in asthma is presumed to be lower than expected compared to available published data (Table 1).

**Table 1:** Prevalence of GERD symptoms in asthmatic patients. GERD: gastroesophageal reflux disease

Reference	Year	Design	Population	Method	Results
Shirai et al. [38]	2015	Cross-sectional study	N = 132 patients with controlled asthma	Questionnaires	Among the 132 subjects with controlled asthma, it was determined that 29 patients (22.0%) had GERD
Bohr et al. [35]	2010	Cross-sectional study	Asthmatics: N = 308; controls: N = 694	Questionnaires	GERD was present in 25.4% of asthmatics vs. 19.4% of controls. Occasional symptoms of GERD present in 21.2% of asthmatics vs. 27.0% of controls
Havemann et al. [12]	2007	Systematic review	28 epidemiological studies	Description of GERD symptoms, severity, frequency, and 24-hour pH-monitoring	The average prevalence of GERD symptoms was 59.2%, compared to 38.1% in controls
Gislason et al. [37]	2002	Cross-sectional study	Total population: N = 2661	Questionnaires and detailed interviews	Asthmatics (n=459) had nocturnal GERD and esophagitis in 40% of cases vs. random population sample (n=101) 4.6% of cases
Field et al. [36]	1996	Cross-sectional study	Asthmatics: N = 109; control groups: N = 68, N = 67	Questionnaires	Among the asthmatics, 77% had heartburn, 55% had regurgitations, and 24% had difficulty swallowing

Pharmacologic management of GERD in asthma 

Considering that a relationship between GERD and asthma has been shown, it is reasonable to assume that treating GERD in asthmatics may be beneficial [3]. In roughly 70% of patients, anti-reflux medication improves asthma-related symptoms (wheezing, coughing, dyspnea, chest discomfort) [1]. Two pharmacologic agents have been described in the literature as an effective therapy for GERD-related symptoms in asthmatic patients; histamine receptor antagonists and proton pump inhibitors.

H2 Receptor Antagonists

Inhibition of the H2 receptor on parietal cells in the stomach mucosa by receptor antagonists like cimetidine or ranitidine leads to reduced gastric acid secretion [3]. Many studies evaluating the effectiveness of these medications were performed in the late 1980s and early 1990s. The majority of studies indicate an improvement in reflux symptoms and, in some cases, recovery from nocturnal asthmatic symptoms. Goodall et al. conducted a double-blind, placebo-controlled, crossover study in 1981 on 20 patients with bronchial asthma plus GERD [39]. Eighteen patients completed the study in which cimetidine 200 mg daily for six weeks was used to control reflux symptoms and later determine positive effects in regards to respiratory function. The outcomes showed an improvement in nocturnal asthma symptoms and peak expiratory flow; however, no change was seen in pulmonary function testing [39]. The effect of ranitidine was evaluated via a double-blind, crossover study by Ekström et al. in 1989 on 48 patients with moderate to severe asthma [40]. The findings concluded that ranitidine 150 mg twice daily for four weeks significantly improved reflux symptoms as well as the modest improvements in nocturnal asthma and daily use of bronchodilators. Exceptionally, no significant variations were found in bronchial reactivity, peak expiratory flow, eosinophil values in blood, and lung function [40]. Notably, Larrain et al. described in their 1991 double-blind, placebo-controlled study of 90 patients with non-allergic asthma a significant decrease in the use of pulmonary medication with the administration of cimetidine 300 mg daily for six months [41]. Improvement in pulmonary function testing was not statistically significant. On the other hand, not all studies have demonstrated alleviation of symptoms. Gustafsson et al. performed a double-blind, crossover, placebo-controlled trial in 1992 for four weeks in 37 children and adolescents with a diagnosis of bronchial asthma and GERD [42]. Ranitidine 300 mg was given as a daily dose (unless weight <40 kg, in which case 150 mg twice daily was used). In individuals with pathological GERD, ranitidine showed a small (30%) but statistically significant decrease in nocturnal asthma symptoms [42]. Similar findings were obtained by Sontag et al. in 2003 via a randomized controlled trial that aimed to study whether long-term treatment of GERD alters the natural history of asthma in patients with coexistent GERD-asthma [43]. A total of 62 patients were included; 24 controls, 22 patients received medical treatment with ranitidine 150 mg three times daily, and 16 received surgical treatment with Nissen fundoplication. Patients were followed up for two years, by which time improvement, notable improvement, or cure in overall asthma status happened in 74.9% of the surgical group, 9.1% of the medical group, and 4.2% of the control group (Table 2). The surgical group's mean asthma symptom score improved by 43%, compared to less than 10% in the medical and control groups (p = 0.0009). In summary, medical therapy with ranitidine did not improve pulmonary function, medication requirements, survival, or improvement of asthma symptoms [43]. 

**Table 2:** Use of histamine receptor antagonists and outcome on asthma control. GERD: gastroesophageal reflux disease; PFTs: pulmonary function tests

Reference	Year	Design	Population	Method	Results
Sontag et al. [43]	2003	Randomized controlled trial	Total population: N=62; controls: N=24; medical treatment: N=22; surgical treatment: N=16	Ranitidine 150 mg three times daily with follow-up in two years	Improvement in asthma status in 74.9% of the surgical group, 9.1% medical group, and 4.2% control group
Gustafsson et al. [42]	1992	Double-blind, crossover, placebo-controlled trial	N = 37 children and adolescents with a diagnosis of bronchial asthma and GERD	Ranitidine 300 mg once daily if weight <40 kg, 150 mg twice daily was used) for four weeks	Ranitidine showed a small (30%) but statistically significant decrease in nocturnal asthma symptoms
Larrain et al. [41]	1991	Double-blind, placebo-controlled study	N = 90 patients with non-allergic asthma	Cimetidine 300 mg daily for six months	Significant decrease in the use of pulmonary medication. Improvement in pulmonary function testing was not statistically significant
Ekström et al. [40]	1989	Double-blind, crossover, placebo-controlled study	N= 48 patients with moderate to severe asthma	Ranitidine 150 mg twice daily for four weeks	Improvement of reflux symptoms, nocturnal asthma, and use of bronchodilators. No significant variations in PFTs
Goodall et al. [39]	1981	Double-blind, placebo-controlled, crossover study	N = 20 patients with bronchial asthma plus GERD	Cimetidine 200 mg daily for six weeks	Improvement in nocturnal asthma symptoms and peak expiratory flow. No change in PFTs

Proton Pump Inhibitors

Proton pump inhibitors (PPIs) are an innocuous and relatively well-tolerated family of medications that directly inhibit the proton pump (H+/K+ ATPase) of the parietal cells in the stomach leading to the suppression of acid [3]. PPIs are referred to as the current gold standard in the treatment of GERD because they provide a demonstrable advantage over histamine blockers in terms of GERD symptom alleviation and esophagitis [3]. Many trials have been conducted to establish the beneficial effect of PPIs on asthma symptoms and pulmonary function tests. However, their advantage has not been clearly shown and remains obscure. Meier et al. performed a double-blind, placebo-controlled, crossover study in 1994 to determine the outcome of reflux suppression with Omeprazole 20 mg twice daily on pulmonary function in asthmatics with esophagitis [44]. Results showed that in four of 15 (27%) patients with asthma and GERD, there was a net improvement of more than or equal to 20% in FEV1 after receiving treatment for six weeks with PPI [44]. Similar findings were obtained by Shimizu et al. in a randomized prospective study conducted in 2006 by evaluating the efficacy of a histamine blocker (roxatidine, 150 mg daily) and a PPI (lansoprazole, 30 mg daily) on asthma symptoms in 30 asthmatic patients with GERD [45]. Lansoprazole, in contrast to roxatidine, greatly improved peak expiratory flow. Nevertheless, neither lansoprazole nor roxatidine led to a change in pulmonary function of FEV1 in patients with asthma [45]. Additionally, a randomized, double-blind, placebo-controlled study led by Kiljander et al. in 2010 investigated the effect of esomeprazole 40 mg once or twice daily in asthmatic patients with associated GERD [46]. A total of 828 patients completed the study during 26 weeks, with findings leading to improvements in peak expiratory flow observed in both esomeprazole 40mg once daily vs. 40 mg twice daily with no statistically significant differences between treatments. Likewise, both doses of esomeprazole improved FEV1 and led to higher scores on questionnaires pertaining to asthma quality of life [46]. Contrary to the precedent findings, not all studies have detailed improvements in asthma symptoms with the use of PPIs (Table 3).

**Table 3:** Use of proton pump inhibitors and outcome on asthma control. PEF: peak expiratory flow; FEV1: forced expiratory volume in the first second; FVC: forced vital capacity; PFTs: pulmonary function tests

Reference	Year	Design	Population	Method	Results
Kiljander et al. [46]	2010	Randomized, double-blind, placebo-controlled study	N = 828 asthmatic patients with associated GERD	Esomeprazole 40 mg once or twice daily during 26 weeks	Improvements in PEF and FEV1 were observed in both groups, with no statistically significant differences between treatments
Mastronarde et al. (American Lung Association Asthma Clinical Research Centers) [47]	2009	Randomized, double-blind, placebo-controlled trial	N = 412 patients with poor asthma control and GERD	Esomeprazole 40 mg twice daily or placebo. Follow-up was performed at 24 weeks	Poor asthma control in both groups (2.3 and 2.5 episodes per person-year). No treatment benefit regarding PFTs or quality of life
Shimizu et al. [45]	2006	Randomized, controlled, prospective trial	N= 30 asthmatic patients with GERD	Roxatidine 150 mg daily vs. lansoprazole 30 mg daily	Lansoprazole, in contrast to roxatidine, greatly improved PEF. Neither lansoprazole nor roxatidine led to a change in FEV1
Littner et al. [48]	2005	Multicenter, double-blind, randomized, placebo-controlled trial	N= 207 patients with moderate to severe persistent asthma with GERD	Lansoprazole 30 mg twice daily or placebo. Follow up at 24 weeks	Lansoprazole did not improve daily asthma symptoms, albuterol usage, PEF, FEV1, FVC. Improvement in asthma exacerbations and quality of life
Meier et al. [44]	1994	Double-blind, placebo-controlled crossover study	N= 15 patients with asthma and GERD	Omeprazole 20 mg twice daily for six weeks	Net improvement of more than or equal to 20% in FEV1 in 27 % of patients (4/15)

This was evidenced by Littner et al. in a multicenter, double-blind, randomized, placebo-controlled trial in 2005 to determine if PPIs improve the control of asthma in adults with concomitant reflux symptoms [48]. Two hundred seven patients with moderate to severe persistent asthma were included and received treatment with lansoprazole 30 mg twice daily or placebo. At 24 weeks, Lansoprazole medication did not substantially improve daily asthma symptoms, Albuterol usage, peak expiratory flow, FEV1, FVC, or investigator-assessed asthma symptoms as compared to placebo. On the other hand, this dosage dramatically decreased asthma exacerbations and improved asthma quality of life, especially in individuals on several asthma medications [48]. An equivalent study displayed similar outcomes in asthma control. The American Lung Association Asthma Clinical Research Centers carried out a randomized double-blind control trial in 2009 where 412 patients with poor asthma control received either esomeprazole 40 mg twice a day or placebo [47]. Follow-up was performed at 24 weeks. The esomeprazole and placebo groups had a comparable number of episodes of poor asthma control (2.5 and 2.3 episodes/person-year; p = 0.66). Individual components of episodes of poor asthma control, as well as secondary outcomes such as pulmonary function, airway reactivity, asthma control, symptom ratings, nocturnal awakening, and quality of life, showed no treatment benefit [47]. Conclusively, patients with poor asthma control do not show improvement despite strong PPIs, which could be related to the lack of GERD exacerbating their symptoms [18]. However, improvement is seen relating to asthma exacerbations and pulmonary function in individuals with symptomatic reflux. If GERD is suspected as a contributing element to patients' persistent asthma exacerbations, most experts concur with an empiric trial with PPIs [18]. The findings of these studies can be visualized below (Table 3).

Limitations

This study does not take into consideration due to various key factors like obesity, chronic obstructive pulmonary disease, genetic susceptibility, atypia, and other environmental features that may present as confounding variables for the interplay between asthma and GERD. Additionally, when reviewing the effectiveness of treatment, no optimal regimen was taken into consideration due to the fact that none has been defined in this subset of patients with respiratory symptoms. Lastly, studies conducted in adults, children, and animal trials were included in this review article without exclusion of a specific population group. 

## Conclusions

Various studies have been conducted over the decades to provide evidence for a causal link between GERD and asthma. In summary, this review article aimed to provide insight into a shared nexus between these two entities based on shared pathophysiology, clinical correlation, and pharmacological management. We believe that this is a relevant topic of discussion due to the fact that asthmatic patients benefit from therapy with PPIs, and clinicians may benefit from the knowledge behind the shared mechanisms of these two disease processes. The pathophysiology of GERD-induced asthma is still heavily debated and under investigation, but the association is clear due to the prevalence of respiratory symptoms among the GERD population. However, evidence is still lacking. Pharmacologic therapy with PPIs is considered the mainstay of treatment for GERD in asthmatic patients and studies have shown that these agents improve certain lung function parameters as well as the quality of life. Lastly, we highly recommend that additional studies in the form of randomized controlled trials be performed to solidify the relationship between GERD and asthma in order to effectively provide guidelines for treatment in this subset of patients.
